# Molecular characterization and antimicrobial susceptibility profiles of Thai *Mycoplasma synoviae* isolates

**DOI:** 10.1038/s41598-023-29266-9

**Published:** 2023-02-03

**Authors:** Kriengwich Limpavithayakul, Jiroj Sasipreeyajan, Somsak Pakpinyo

**Affiliations:** Avian Health Research Unit, Department of Veterinary Medicine, Faculty of Veterinary Science, Chulalongkorn University, Pathumwan, Bangkok, 10330 Thailand

**Keywords:** Microbiology, Molecular biology, Diseases

## Abstract

*Mycoplasma synoviae* (MS) infection is mainly controlled by pathogen-free flocks’ maintenance, medication in infected flocks, and vaccination in high-risk flocks. The effective control strategy requires convenient approach for detecting and differentiating MS strains and reliable drug susceptible evidence for deciding on reasonable antimicrobial usage. This study aimed to characterize the partial *vlhA* gene of nine Thai MS isolates circulated in chickens in 2020, to verify the PCR-RFLP assay for strain differentiation, and to determine the eight antimicrobial susceptibility profiles using microbroth dilution method. Based on sequence analysis of the partial *vlhA* gene, Thai MS isolates in 2020 were classified as types E and L with 19 and 35 amino acid lengths, respectively. The developed PCR-RFLP assay could detect and differentiate vaccine and Thai field strains. Most Thai MS isolates in this study were susceptible to tylosin, tylvalosin, tiamulin, doxycycline, oxytetracycline, tilmicosin, and lincomycin-spectinomycin at MIC_50_ values of 0.0391, 0.0098, 0.0781, 0.1563, 0.1563, 0.625 and 0.625 μg/mL, respectively; and resistance to enrofloxacin at MIC_50_ value of 10 μg/mL. In conclusion, this study revealed diagnostic assays for differentiating MS strains and the antimicrobial susceptibility profiles of Thai MS*,* which are necessary to design suitable MS control procedures for poultry flocks.

## Introduction

*Mycoplasma synoviae* infection, an economic consequence disease in poultry industry worldwide, predisposes airsaculitis, lameness, carcasses’ condemnation, and poor eggshell quality. The most effective disease control strategy; *M. synoviae*-free flocks’ maintenance with single-age management and infected flock elimination; is not economic compliance in poultry business; therefore, medication in infected flocks and vaccination in high-risk flocks have been suggested as alternative measures representing an apparent efficacy in reducing clinical signs or improving production performance in poultry industrial level^1–9^. For decades, medication has been more common practice than vaccination because of the limited countries having available live vaccine registration, while vaccination with commercial live attenuated MS-H vaccine, a temperature-sensitive strain, has been dramatically increasing in several countries challenged with raising awareness of antimicrobial resistance. Although an attractive optimal cost-benefit relation is exhibited in medication and vaccination, medication cannot completely eliminate *M. synoviae* infection from affected breeder or layer chicken flocks, whereas vaccination is questionable in disease control and eradication programs^3–8,10^.

Medication in infected flocks; using mycoplasma-susceptible antimicrobials inhibiting protein synthesis, including tetracyclines (oxytetracycline, chlortetracycline, and doxycycline), macrolides (tylosin, tilmicosin, tylvalosin, erythromycin, and spiramycin), lincosamides (lincomycin), quinolones (enrofloxacin), and pleuromutilins (tiamulin); could reduce antimicrobial use in the next and the progeny flocks but the long intensive treatment is not generally acceptable^5,6,8,11^. Moreover, the current antimicrobial resistance issue has influenced the practical use of antimicrobials for controlling *M. synoviae* infection in poultry flocks, including the requirement of prescription from poultry veterinarian and the reliable susceptible profiles like minimum inhibitory concentration (MIC) of *M. synoviae* field isolates^12,13^.

The diagnostic assays have been developed based on the polymerase chain reaction (PCR) assays and sequence analysis^4,14–19^. However, the information of diagnostic assays for differentiating MS-H vaccine strain from *M. synoviae* field strains/isolates is limited^4,20^. The 16S rRNA gene-based PCR assay is mostly used for detecting *M. synoviae* organisms, while the *vlhA* gene-based PCR assay and sequence analysis are suitable for differentiating *M. synoviae* strains^4,14–19^. Consequently, based on sequence analysis of partial *vlhA* gene of *M. synoviae* worldwide, Thai *M. synoviae* field isolates were identified as types C (isolated from cloanal cleft), E (isolated from cloanal clefts and articular joints), and L (isolated from cloanal clefts, articular joints and yolk sac membrane) which could differentiate from MS-H vaccine strain^14,16,21,22^, but sequence analysis is considered a time-consuming assay^4,16,18,22^. Therefore, the PCR-restriction fragment length polymorphism (PCR-RFLP) assay, using the restriction enzyme TasI to digest *vlhA* gene-targeted PCR product, has been developed to become the convenient assay with high sensitivity and specificity for detecting and differentiating MS-H vaccine from non-vaccine strains circulating in Thailand.

Accordingly, this study aimed to characterize the partial *vlhA* gene of *M. synoviae* isolates recently circulated in Thailand by sequence analysis and to verify the PCR-RFLP assay as a convenient approach for strain differentiation. In addition, to fulfill the missing information in medication, this study was also conducted to determine the antimicrobial susceptibility profiles by using microbroth dilution method with common antimicrobials used in veterinary practice against avian mycoplasmosis including enrofloxacin, oxytetracycline, doxycycline, tiamulin, tylosin, tilmicosin, tylvalosin and lincomycin-spectinomycin.

## Materials and methods

### Isolation of *M. synoviae* field isolates

*M. synoviae* field isolates were obtained from approximately ninety flocks of registered commercial chicken farms, including breeder flocks, broiler flocks, and layer flocks in Thailand in 2020. Chickens were individually swabbed at the articular joint or the respiratory tract; choanal cleft, tra,chea and airsac; using a sterilized cotton swab. Each swab sample was identified and inoculated into 2 mL of Frey’s broth medium supplemented with 15% swine serum (FMS broth)^23^. The broth samples were then submitted to determine by PCR assays based on the 16S rRNA gene and the *vlhA* gene. The use of experimental animals was approved by Chulalongkorn University Animal Care and Use Committee (IACUC), protocol No.1931051. This study is reported in accordance with ARRIVE guidelines (https://arriveguidelines.org). All methods were performed in accordance with the relevant guidelines and regulations. Good practice principles were respected to minimize the discomfort and provide well-being to chickens.

### *M. synoviae* vaccine, reference, and field strains

The live *M. synoviae* vaccine; Vaxsafe MS® (Bioproperties, Australia); is MS-H strain which is the temperature-sensitive strain developed in Australia since 1996 and introduced in Thailand since 2012. The reference strains of *M. synoviae* and *M. gallisepticum*, used as positive and negative controls in PCR assays, were *M. synoviae* WVU 1853 strain and *M. gallisepticum* S6 strain, respectively. The current nine Thai *M. synoviae* isolates, identified as types E and L, and Thai *M. synoviae* field isolates previously identified as type L in 2015^16^ were also included in this study.

*M. synoviae* MS-H vaccine, *M. gallisepticum* S6, *M. synoviae* WVU 1853, and Thai *M. synoviae* field strains were cultured in 2 mL of FMS broths and subsequently analyzed by PCR assays. An appropriate risk assessment was approved by Institution Biosafety Committee (CU-VET-BC), protocol No. IBC1931053.

### Culture method

FMSbroth samples were incubated at 37 °C in a humidified chamber for 5–7 days until the broth color changed from pink-red to orange-yellow. Then, the broth samples were divided into two portions. The first portion was subjected to extract the DNA for *M. synoviae* specific PCR assay, 16S rRNA gene-based PCR assay. The remaining portion was immediately diluted for culture on FMS agar and incubated at 37 °C in humidified condition before sampling a single colony of *M. synoviae* isolate. Consequently, five selected single *M. synoviae* colonies were passaged into fresh FMS broth and incubated at 37 °C in humidified condition until the broth color changed from pink to orange-yellow. The FMS cultured broth, showing mycoplasma growth, was then equally divided into three portions. The first portion was extracted the DNA for the 16S rRNA gene and the *vlhA* -based PCR assays. The second and third portions were stored at − 80 °C as frozen stock of each pure *M. synoviae* isolate for further study^14^.

### DNA templates preparation

DNA was extracted from FMS broth samples using the modified rapid boiling DNA extraction^24^. Broth samples were centrifuged at 16,000 × g for 6 min, washed two times with sterile phosphate-buffered saline (PBS), and resuspended in 50 µl of sterile PBS. The suspended cell was boiled at 100 °C for 10 min, placed on ice for 10 min, and centrifuged at 16,000 × g for 6 min. The supernatant containing DNA template was collected and stored at -20 °C until used. Concentration of the DNA template was determined by using a NanoDrop™ Spectrophotometer.

### *M. synoviae*-specific PCR assay

DNA templates were examined by the Lauerman 16S rRNA gene-based PCR assay^15^. PCR mixture 50 µl contained 35 µl of nuclease-free distilled water, 5 µL of 5 × Green GoTaq^®^ Flexi Buffer (Promega, Madison, WI, USA), 2.5 µL of 25 mM MgCl_2_, 1 µL of 10 mM dNTP (Fermentas, Leon-Rot, Germany), 0.5 µL of each 10 µM primer MSL-1 (5'-GAA GCA AAA TAG TGA TAT CA-3') and primer MSL-2 (5'-GTC GTC TCC GAA GTT AAC AA-3') (Qiagen^®^, Valencia, CA, USA), 0.5 µL of 5 U/µL GoTaq^®^ Flexi DNA Polymerase (Promega, Medison, WI, USA), and 5 µL of DNA template 100–200 ng. *M. gallisepticum* S6 strain and *M. synoviae* WVU 1853 strain were used as negative and positive controls, respectively. The PCR mixtures were amplified in a DNA thermal cycler (Life express, BIOER^®^, ROC) starting with 94 °C for 5 min and 40 cycles of 94 °C for 1 min, 55 °C for 1 min, and 72 °C for 2 min and then followed by 72 °C for 5 min at the final extension. The PCR products were analyzed using gel electrophoresis.

### PCR amplification of partial *vlhA* gene

The *vlhA* gene fragment of *M. synoviae* positive samples was amplified using the revised Hammond *vlhA* gene-targeted PCR assay^17^. The 50 µl PCR mixture contained 34 µl of nuclease-free distilled water, 5 µL of 5 × Green GoTaq^®^ Flexi Buffer (Promega, Madison, WI, USA), 2.5 µL of 25 mM MgCl_2_, 1 µL of 10 mM dNTP (Fermentas, Leon-Rot, Germany), 1 µL of each 10 µM primer MSRH-1 (5'- GGC CAT TGC TCC TRC TGT TAT -3') and primer MSRH-2 (5'- AGT AAC CGA TCC GCT TAA TGC -3') (Qiagen^®^, Valencia, CA, USA), 0.5 µL of 5 U/µL GoTaq^®^ Flexi DNA Polymerase (Promega, Medison, WI, USA), and 5 µL of DNA template 100–200 ng. *M. gallisepticum* S6 strain and *M. synoviae* WVU 1853 strain were used as negative and positive controls, respectively. The PCR mixtures were amplified in a DNA thermal cycler (Life express, BIOER^®^, ROC) starting with 95 °C for 3 min and 40 cycles of 94 °C for 1 min, 56 °C for 1 min, and 72 °C for 1 min and then followed by 72 °C for 5 min at the final extension. The PCR products were analyzed using gel electrophoresis.

### Sequence analysis of partial *vlhA* gene

The *vlhA* gene PCR products from the revised Hammond *vlhA* gene-targeted PCR assay containing *vlhA* DNA fragments were purified and subjected to sequencing at A T G C Co. Ltd. (Thailand Science Park, Pathum Thani, Thailand). A similarity of nucleotide sequence was analyzed using the BLAST program (www.ncbi.nlm.nih.gov/BLAST). Sequencing alignment analyses, corresponding to the N-terminal *vlhA* gene of *M. synoviae* K1968 strain classified as type B, were performed using the molecular evolutionary genetic analysis (MEGA 10) software (http://www.megasoftware.net). *M. synoviae* isolates were typed based on the description of the proline-rich repeat (PRR) region of *vlhA* gene. *M. synoviae* types A, B, C, D, E, F, G, H, I, J, K, and L were classified based on the length of PRR fragments of 38, 45, 32, 23, 19, 36, 51, 46, 28, 20, 12 and 35 amino acids, respectively^14,16,21,22,25^.

### PCR-RFLP assay

The PCR-RFLP assay was developed by using the restriction enzyme map analysis tool on the Genescript webpage (https://www.genscript.com/tools/restriction-enzyme-map-analysis) to reveal the restriction enzyme TasI (ThermoFisher Scientific, San Jose, CA, USA), cutting best at 65 °C in B buffer, as the suitable restriction enzyme which could digest *vlhA* gene-targeted PCR products of *M. synoviae* positive samples. Briefly, the 20 µL of *vlhA* gene-based PCR product containing DNA at least 0.05 µg/µL were added to the 23 µL of TasI mixture containing 4 µL of 10 × Buffer B, 1 µL of TasI (10 U/ µL; ThermoFisher Scientific, San Jose, CA, USA) and 18 µL of nuclease-free distilled water. *M. gallisepticum* S6 strain and *M. synoviae* WVU 1853 strain were used as negative and positive controls, respectively. After incubation at 65 °C for 2 h, the digested PCR products were separated and analyzed using gel electrophoresis.

### Gel electrophoresis

The PCR products resulted from PCR and PCR-RFLP assays were analyzed in 2% agarose gel (Vivantis Technologies, Malaysia) in 1 × TBE buffer at 100 V for 35 min, pre-stained with MaestroSafe™ dye (Maestrogen, Las Vegas, NV, USA), visualized by UV transilluminator, and photographed. The amplicon size was compared to standard 100 bp DNA ladder (New England Biolab, UK).

### Tested antimicrobials

Tested antimicrobials using in this study were registered and approved by the Food and Drug Administration, Ministry of Public Health, Thailand. Eight tested antimicrobials selected for determining the antimicrobial susceptibility profiles in this study were common antimicrobials used in commercial farm in Thailand including enrofloxacin (Poren^®^ containing 200 mg/mL; Seven Stars Pharmaceutical, Samphran, Nakorn Prathom, Thailand), oxytetracycline hydrochloride (Terramycin™ containing 200 mg/mL; Zoetis (Thailand) Co., Ltd.), doxycycline hyclate (Doxine 500 WSP containing 500 mg/g; Better Pharma Co., Ltd., Lak Si, Bangkok, Thailand), tiamulin hydrogen fumarate (Denagard™ containing 450 mg/g; Elanco (Thailand) Co., Ltd.), tylosin tartrate (Tysol containing 860 mg/g; NutriChems Co., Ltd., Kaeng Khoi, Saraburi, Thailand), tilmicosin phosphate (Pulmotil™ AC containing 250 mg/mL; Elanco (Thailand) Co., Ltd.), tylvalosin tartrate (Aivlosin^®^ containing 625 mg/g; ECO company, UK) and lincomycin-spectinomycin (Linco-Spectin™ 100 containing lincomycin hydrochloride 222 mg/g and spectinomycin sulphate 445 mg/g; Zoetis (Thailand) Co., Ltd.).

### Determination of antimicrobial susceptibility profiles

A viable count of *M. synoviae* isolates; WVU 1853 strain, nine field isolates in 2020, and a field isolate in 2015; in color changing unit (CCU) was obtained using the most probable number (MPN) determined using an MPN table^26,27^. Briefly, 20 μL of each *M. synoviae* isolate culture broth from frozen stocks was filled into each well of the 1st column of the 96-well plate containing 180 μL of FMS broth and then serially tenfold diluted from the 1st until the 11th column. Besides, the 12th column contained only the 200 μL of fresch FMS broth. Consequently, each cultured plate was incubated at 37 °C in humidified condition for 14 days before the number of wells in the last three columns showing the color changing from red to yellow was counted and estimated the CCU.

The frozen stocks of *M. synoviae* isolates were used as inocula for evaluating the in vitro susceptibility to eight antimicrobials, including enrofloxacin, oxytetracycline, doxycycline, tiamulin, tylosin, tilmicosin, tylvalosin, and lincomycin-spectinomycin. Besides, the tested antimicrobials were formulated and diluted in FMS broth. Antimicrobial susceptibility profiles were determined by final MIC values using serial broth dilution method^28^. Briefly, duplicate wells of antimicrobials were two-folded, serially diluted in a 100 µL of FMS broth in the sterile 96-well, flat-bottomed microtitration plates. The 100 µL of FMS broth containing *M. synoviae* organisms approximately 10^5^ CCU/mL was added to each well from the 1^st^ until the 11^th^ column containing the same amount of antimicrobial at final concentrations 10, 5, 2.5, 1.25, 0.625, 0.3125, 0.1563, 0.0781, 0.0391, 0.0195 and 0.0098 µg/mL. Positive control consisting of only *M. synoviae* cultured broth was included at the 12th column in each plate.

The MIC values were recorded daily after the positive control broth color changed, and the final MIC values were assessed at 14 days after incubation. The lowest concentration of each antimicrobial that completely prevented the broth color changing from pink to orange-yellow was considered as MIC. The MIC_50_ and MIC_90_ values were defined as the lowest concentrations that inhibited the growth of 50% or 90% of the strains, respectively. The MIC breakpoints of avian mycoplasmas for eight tested antimicrobials were based on the previous MIC determination (Table 1)^5,11,29,30^.

**Table 1:** MIC breakpoints of the eight tested antimicrobials^5,11,29,30^.

Antimicrobial	Susceptible (µg/ml)	Intermediate (µg/ml)	Resistant (µg/ml)
Enrofloxacin	≤ 0.5	1	≥ 2
Oxytetracycline	≤ 4	8	≥ 16
Doxycycline	≤ 4	8	≥ 16
Tiamulin	≤ 1	2	≥ 4
Tylosin	≤ 1	2	≥ 4
Tilmicosin	≤ 1	2	≥ 4
Tylvalosin	≤ 1	2	≥ 4
Lincomycin-spectinomycin	≤ 1	2	≥ 4

### Statistical analysis

Comparison of MIC values between Thai *M. synoviae* field isolates, which were classified as types E and L or originated from articular joint and respiratory tract, was analyzed by using independent samples t-test. Statistical analyses were performed using IBM SPSS Statistics 22 windows and differences were considered as significant at *P* < 0.05.

## Results

### Sequence analysis of partial *vlhA* gene

*M. synoviae* field isolates obtained from registered chicken farms in 2020 were confirmed by the 16S rRNA gene-based PCR assay and were then selected for sequence analysis of partial *vlhA* gene. The nucleotide sequences were submitted to the GenBank Database. Details, including sizes of amplicons, PRRs nucleotide and amino acid sequences, and GenBank accession number, were shown in Table 2.

**Table 2:** Information and molecular characteristic details of *M. synoviae* isolates and strains used in this study.

Isolates ID	Source of MS^A^	Age (weeks)	Province	Sequence analysis of partial *vlhA* gene-			GenBankAccession no
				PCR amplicons (bp)	PRR length (nt / aa)	MS type	
WVU1853 strain (ATCC 25,204)	Reference strain			376	114/38	A	KX168667, AM998371, ON191513
MS-H strain (Australia origin)	Vaccine strain			358	96/32	C	KX168666, JX960401, ON191514
AHRU2020CK0615	Broiler / C	4	Prachin Buri	319	57/19	E	ON191515
AHRU2020CK0301	Layer / J	19	Chon Buri	319	57/19	E	ON191516
AHRU2020CK0305	Layer / J	19	Chon Buri	319	57/19	E	ON191517
AHRU2020CK0404	Layer / J	22	Chon Buri	319	57/19	E	ON191518
AHRU2020CK0709	Layer / J	16	Chon Buri	319	57/19	E	ON191519
AHRU2020CU1401	Layer / C	60	Chachoengsao	367	105/35	L	ON191520
AHRU2020CU1409	Layer / C	60	Chachoengsao	367	105/35	L	ON191521
AHRU2020CU1505	Broiler / Y	1	Prachin Buri	367	105/35	L	ON191522
AHRU2015CU2802^B^	Native broiler / J	9	Satun	367	105/35	L	KX168690, ON191523
AHRU2018CK0301	Layer / J	15	Chon Buri	319	57/19	E	ON191524
AHRU2020CU1104	Layer / C	55	Chon Buri	319	57/19	E	ON191525
AHRU2020CU1101	Layer / C	55	Chon Buri	319	57/19	E	ON191526
AHRU2020CU1323	Layer breeder / C	7	Nakhon Nayok	358	96/32	C	ON191527
AHRU2020CK1206	Layer breeder / C	22	Chaiyaphum	367	105/35	L	ON191528
MS-1 strain (NAD-independent)	Vaccine strain			376	114/38	A	ON191529

^**A**^Abbreviations C, J, and Y represented the swab samples from choanal cleft, synovial fluid, and yolk sac.

^**B**^AHRU2015CU2802 is a Thai *M. synoviae* field strain isolated in 2015.

According to sequence analyses of partial *vlhA* gene, the PRR type of *M. synoviae* WVU 1853 and MS-H vaccine strains were identified as groups A and C, respectively. Besides, most Thai *M. synoviae* isolates in 2020 were identified as types E and L with 19 and 35 amino acid lengths, respectively. Five Thai *M. synoviae* isolates collected from articular joint were classified as type E, while Thai field isolates from respiratory tract were classified as types E (3 isolates) and L (4 isolates).

### PCR-RFLP assay

**Figure 1:**
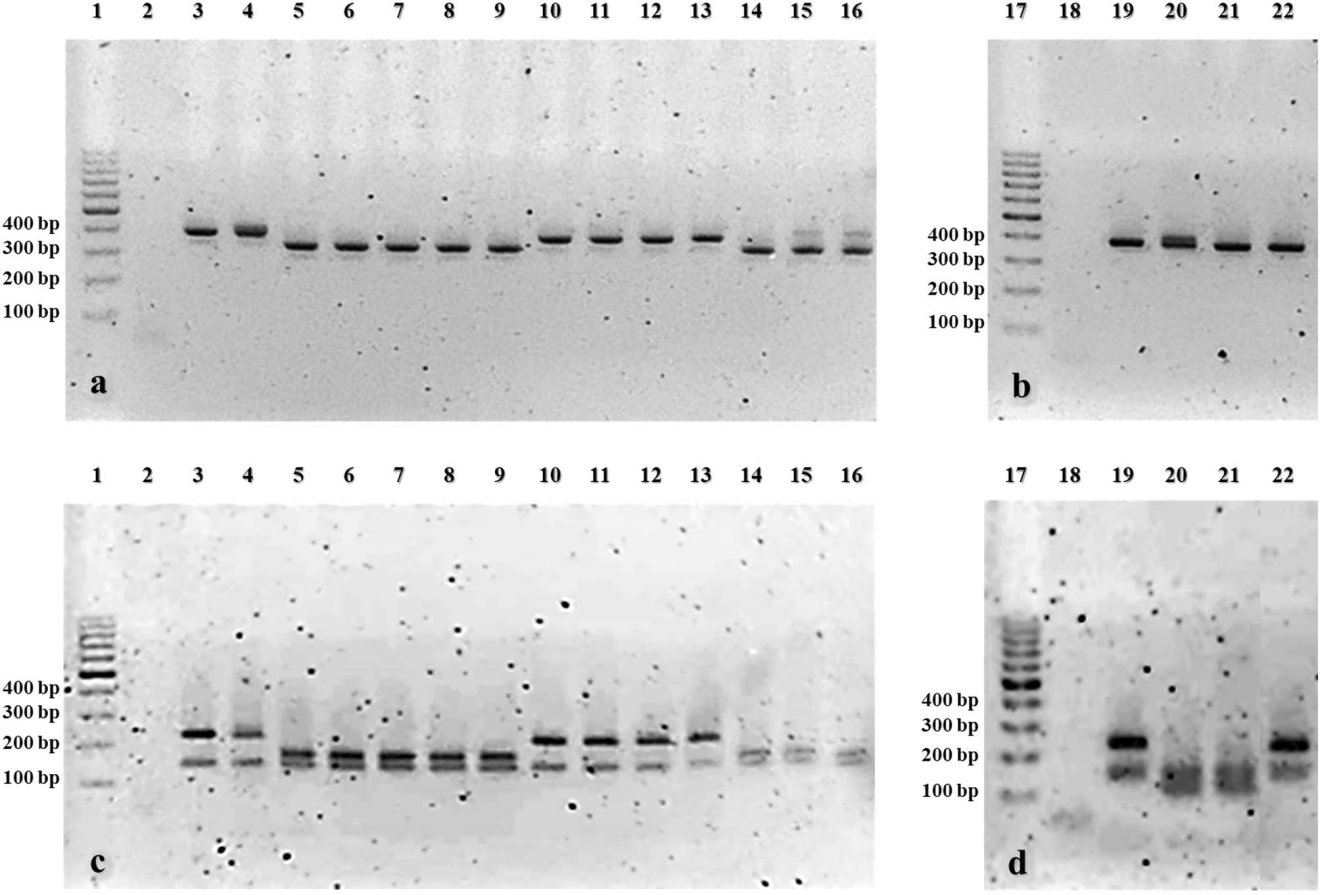
Electrophoresis gel demonstrating PCR products from *M. synoviae* isolates consisting of the full length of partial *vlhA* gene amplicons size of 350–400 bp (**a**, **b**) and the two digested fragments of partial *vlhA* gene amplicons size of 100–200 bp (**c**, **d**). Lane 1 and 17, 1000 bp DNA ladder; Lane 2 and 18, *M. gallisepticum* S6 strain as negative control; Lane 3 and 19, *M. synoviae* WVU 1853 strain as positive control; Lane 4, *M. synoviae* MS-1 strain; Lane 5, AHRU2020CK0615; Lane 6, AHRU2020CK0301; Lane 7, AHRU2020CK0305; Lane 8, AHRU2020CK0404; Lane 9, AHRU2020CK0709; Lane 10, AHRU2020CU1401; Lane 11, AHRU2020CU1409; Lane 12, AHRU2020CU1505; Lane 13, AHRU2015CU2802; Lane 14, AHRU2018CK0301; Lane 15, AHRU2020CU1104; Lane 16, AHRU2020CU1101; Lane 20, *M. synoviae* MS-H vaccine strain; Lane 21, AHRU2020CU1323; Lane 22, AHRU2020CK1206.

The PCR-RFLP assay was developed and validated with *M. synoviae* field isolates recently circulating in Thailand. PCR amplicons of partial *vlhA* gene in Fig. 1; full-length amplicons size 350–400 bp (Fig. 1a,b) and two digested fragments size 100–250 bp (Fig. 1c,d); were presented specific two digested fragments size 100 and 134 bp of *M. synoviae* MS-H strain (Fig. 1d, Lane 20) in Thai *M. synoviae* isolate AHRU2020CU1323 (Fig. 1d, Lane 21) which were different from other *M. synoviae* non-vaccine isolates; *M. synoviae* WVU 1853 strain (Fig. 1c, Lane 3 and Fig. 1d, Lane 19) and Thai *M. synoviae* field strains (Fig. 1c, Lanes 5–16 and Fig. 1d, Lane 22); which concisely presented the specific two digested fragments size 130 and 160–210 bp. The original version of electrophoresis gel with membrane edges visible demonstrating PCR products from *M. synoviae* isolates were presented in Supplementary Fig. 1.

### Antimicrobial susceptibility profiles

MIC values of tested antimicrobials; enrofloxacin, doxycycline, oxytetracycline, tylosin, tilmicosin, tylvalosin, lincomycin in combination with spectinomycin, and tiamulin; against Thai *M. synoviae* field and reference strains were shown in Tables 3 and 4. *M. synoviae* WVU 1853 strain and Thai *M. synoviae* isolate AHRU2020CU1323 (or MS-H like strain) were presented low final MIC values of tylosin, tilmicosin, and tylvalosin at 0.0098 μg/mL and high final MIC values of enrofloxacin and lincomycin-spectinomycin at 0.3125 and 0.625 μg/mL, respectively. Thai *M. synoviae* isolates AHRU2020CU1323 and AHRU2020CU1409 were respectively showed the lowest final MIC value of enrofloxacin (at 0.3125 and 2.5 μg/mL) and oxytetracycline (at 0.0195 and 0.0391 μg/mL). Thai *M. synoviae* field isolates AHRU2020CK0301, AHRU2020CK0305 and AHRU2020CK0404 were performed the high final MIC value of tilmicosin (at 0.625 μg/mL) and tiamulin (at 0.1563 and 0.3125 μg/mL).

**Table 3:** Details of 9 M*. synoviae* isolates in 2020, 1 M*. synoviae* isolate in 2015 and WVU 1853 as *M. synoviae* isolate reference strain and final MIC values of *M. synoviae* isolates.

Isolates ID	Type	MIC values (μg/ml)							
		EFX	DX	OTC	TYL	TIL	TVN	LC-SP	TIA
AHRU2020CK0615	E	10	0.1563	0.1563	0.0391	0.0781	0.0098	0.6250	0.0391
AHRU2020CK0301	E	10	0.1563	0.3125	0.1563	0.6250	0.0195	0.6250	0.1563
AHRU2020CK0305	E	10	0.1563	0.1563	0.0781	0.6250	0.0195	0.6250	0.1563
AHRU2020CK0404	E	10	0.3125	0.3125	0.1563	0.6250	0.0391	0.6250	0.3125
AHRU2020CK0709	E	10	0.3125	0.1563	0.0391	0.0781	0.0098	0.3125	0.0781
AHRU2020CU1409	L	2.5	0.1563	0.0391	0.0098	0.0781	0.0098	0.6250	0.0391
AHRU2020CU1401	L	10	0.1563	0.1563	0.0098	0.0195	0.0098	0.6250	0.0781
AHRU2020CU1505	L	10	0.3125	0.3125	0.0098	0.0195	0.0098	0.6250	0.0781
AHRU2015CU2802	L	10	0.3125	0.3125	0.0781	0.1563	0.0098	0.1563	0.0391
AHRU2020CU1323	C	0.3125	0.0391	0.0195	0.0098	0.0098	0.0098	0.6250	0.0195
WVU1853	A	0.6250	0.1563	0.1563	0.0098	0.0098	0.0098	0.6250	0.0781

Abbreviations of antibiotics: EFX enrofloxacin, DX doxycycline, OTC oxytetracycline, TYL tylosin, TIL tilmicosin, TVN tylvalosin, LC-SP lincomycin-spectinomycin, TIA tiamulin.

**Table 4:** The MIC value range, MIC_50,_ and MIC_90_ of *M. synoviae* isolates in this study.

Antibiotics	MIC values (μg/ml)									
	Reference	MS-H like	MS type E	MS type L	Cleft origin	Joint origin	MS field isolate			
	WVU 1853	AHRU2020CU1323	(n = 5)	(n = 4)	(n = 4)	(n = 5)	(n = 9)			
			Mean ± SE	Mean ± SE	Mean ± SE	Mean ± SE	Mean ± SE	Range	MIC_50_	MIC_90_
EFX	0.625	0.3125	10.00 ± 0.00	8.125 ± 1.875	8.125 ± 1.875	10.00 ± 0.00	9.167 ± 1.157	2.5–10	10	10
DX	0.1563	0.0391	0.219 ± 0.038	0.234 ± 0.045	0.195 ± 0.039	0.250 ± 0.038	0.226 ± 0.031	0.1563–0.3125	0.1563	0.3125
OTC	0.1563	0.0195	0.219 ± 0.038	0.205 ± 0.066	0.166 ± 0.056	0.250 ± 0.038	0.213 ± 0.034	0.0391–0.3125	0.1563	0.3125
TYL	0.0098	0.0098	0.094 ± 0.026	0.027 ± 0.017	0.017 ± 0.007	0.102 ± 0.023	0.064 ± 0.018	0.0098–0.1563	0.0391	0.1563
TIL	0.0098	0.0098	0.406 ± 0.134	0.068 ± 0.032	0.049 ± 0.017	0.422 ± 0.125	0.256 ± 0.087	0.0195–0.6250	0.0781	0.625
TVN	0.0098	0.0098	0.020 ± 0.005	0.010 ± 0.000	0.010 ± 0.000	0.020 ± 0.005	0.015 ± 0.003	0.0098–0.0391	0.0098	0.0195
LC-SP	0.625	0.625	0.563 ± 0.063	0.508 ± 0.117	0.625 ± 0.000	0.469 ± 0.099	0.538 ± 0.053	0.1563–0.6250	0.625	0.625
TIA	0.0781	0.0195	0.148 ± 0.047	0.059 ± 0.011	0.059 ± 0.011	0.148 ± 0.047	0.109 ± 0.028	0.0391–0.3125	0.0781	0.1563

Abbreviations of antibiotics: *EFX* Enrofloxacin, *DX* Doxycycline, *OTC* Oxytetracycline, *TYL* Tylosin, *TIL* Tilmicosin, *TVN* Tylvalosin, *LC-SP* Lincomycin-spectinomycin, *TIA* Tiamulin.

Besides, most Thai *M. synoviae* field isolates showed resistance to enrofloxacin at MIC_50_ value of 10 μg/ml and presented the susceptibility to tylosin, tilmicosin, tylvalosin, and tiamulin at MIC_50_ value of 0.0391, 0.0781, 0.0098 and 0.0781 μg/mL, respectively. Doxycycline, oxytetracycline, and lincomycin-spectinomycin were also performed good activity against Thai *M. synoviae* isolates at a MIC_50_ value of 0.1563, 0.1563, and 0.625 μg/mL, respectively.

Although Thai *M. synoviae* types E and L were insignificantly presented the difference of mean MIC values among each antimicrobial, mean MIC values of tylosin, tilmicosin, tylvalosin, and tiamulin against Thai *M. synoviae* type E seemed to be higher level than Thai *M. synoviae* type L. In addition, mean MIC values of tylosin and tilmicosin against Thai *M. synoviae* isolates originated from respiratory tract were significantly lower than isolates originated from joint (*p*-value of 0.018 and 0.041 for tylosin and tilmicosin, respectively); however, mean MIC values of other antimicrobials were also insignificant lower in respiratory tract origin isolates than joint origin isolates.

Except for enrofloxacin and lincomycin-spectinomycin, MIC_90_ values of doxycycline, oxytetracycline, tylosin, tilmicosin, tylvalosin, and tiamulin were slightly higher than MIC_50_ values. Tylvalosin, tylosin, and tiamulin could perform good susceptibility at MIC_90_ values of 0.0195, 0.1563, and 0.1563 μg/mL, respectively.

**Figure 2:**
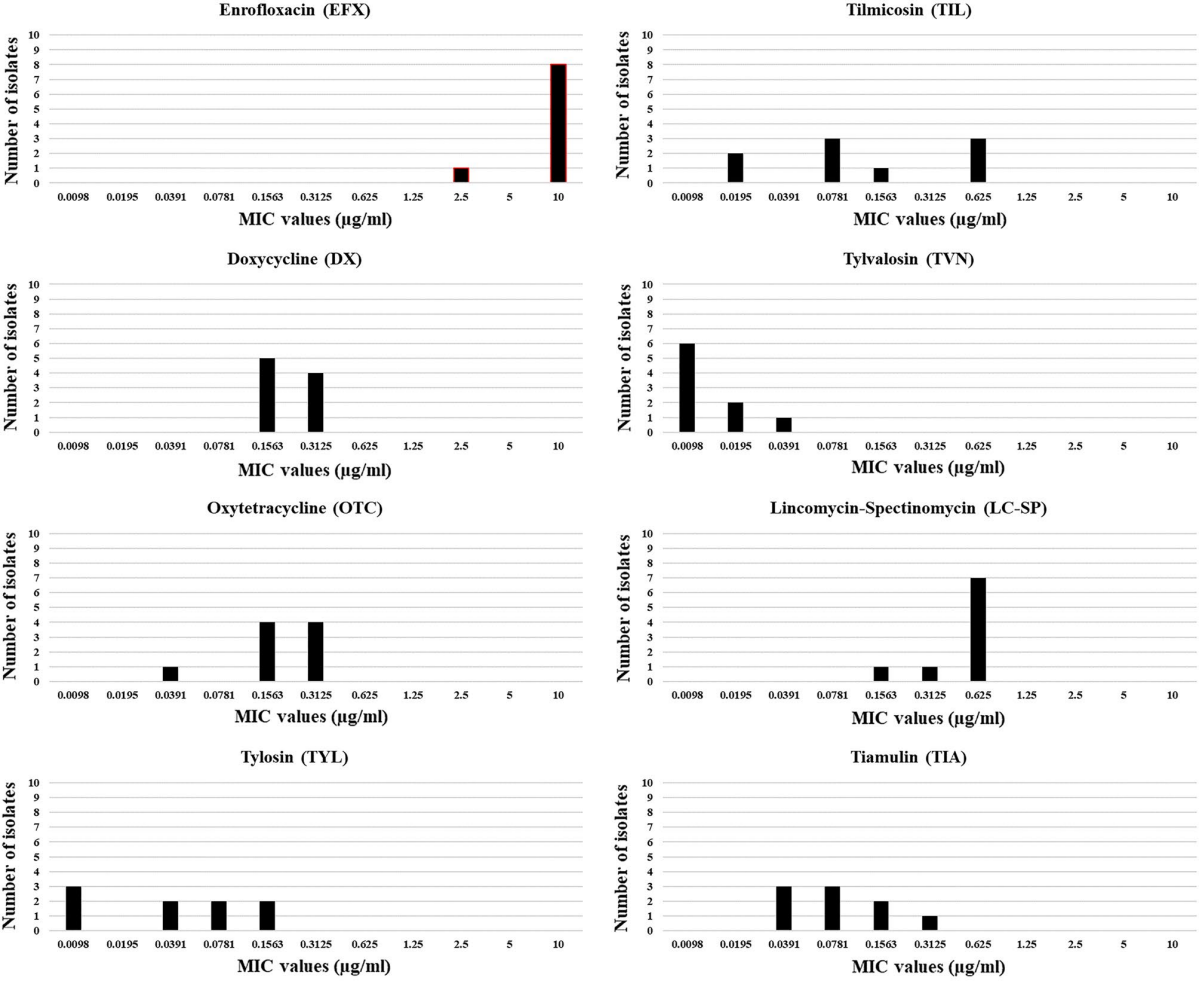
Bar charts illustrate the distribution of *M. synoviae* isolates among the final MIC values of tested antibiotics, including enrofloxacin, doxycycline, oxytetracycline, tylosin, tilmicosin, tylvalosin, lincomycin-spectinomycin, and tiamulin.

In addition, an evidence of subpopulations differed in their MIC values. Most *M. synoviae* field isolates in this study represented one population performing strong susceptible to tylosin, tylvalosin, and tiamulin; moderate susceptible to doxycycline, oxytetracycline, and lincomycin-spectinomycin; and resistance to enrofloxacin. Distribution of *M. synoviae* field isolates in tilmicosin presented at least three subpopulations with different MIC values from strong to moderate susceptibility to tilmicosin (Fig. 2).

## Discussion

This study was conducted to define the genetic characterization of *M. synoviae* isolates currently circulated in Thailand by sequence analysis of partial *vlhA* gene. Based on the PRR region length, Thai *M. synoviae* isolates during 2020 classified as types E and L with 19 and 35 amino acid lengths, respectively; were presented at least two genotypes of current Thai field *M. synoviae* isolates, which differed from the previous study in 2015 showing at least three genotypes and showing Thai *M. synoviae* type L as arthropathic strain^16^. Besides, both Thai *M. synoviae* types E and L were currently isolated from articular joint, and Thai *M. synoviae* type L was isolated from respiratory tract; therefore, both Thai *M. synoviae* types E and L were possibly caused the infection at synovial membrane or respiratory tract, but they might not be associated with oviduct infection, causing eggshell apex abnormality syndrome like Dutch *M. synoviae* isolates types C and E^31^. Furthermore, in addition to joint and respiratory tract, Thai *M. synoviae* field isolates should be further investigated and obtained from the oviduct or reproductive tract especially in the multiple-age layer flocks which always predisposes the emergence of *M. synoviae* virulent strains^4^.

Besides, the experimental inoculation of current Thai *M. synoviae* types E and L via intranasal and intravenous routes in chickens should be further studied to clarify pathogenesis because the pathogenesis of *M. synoviae* infection depends on transmissible routes. An intravenous inoculation and a food pad injection are more frequently causing progressive synovitis lesions, while a respiratory tract inoculation or aerosol exposure usually presents mild synovitis lesions^32,33^. The clarification of pathogenesis, tissue tropism, and internalization properties could be the critical information for understanding the transposition strategy of *M. synoviae* from the mucous barrier to reach more favorable niches which *M. synoviae* could resist to antimicrobials treatment or could evade the host immune response^34^.

Interestingly, in several countries challenged with antimicrobial resistance issue, immunization with live vaccine has been dramatically increasing and is especially proposed to use in risk farms due to possible protection capability against respiratory signs, airsacculitis, egg production losses, and egg transmission^3,4,7,35,36^. Due to the available live commercial vaccine, the MS-H strain which is a temperature-sensitive mutant strain growing well at 33 °C, has been classified as type C with 32 amino acid length of PRR region^9^; vaccination with live MS-H vaccine may present better protection against *M. synoviae* field isolates type C, than types E and L. Therefore, the disappearance of Thai *M. synoviae* field isolates type C in the current surveillance may be the consequence of the protective efficacy of live MS-H vaccine to control and reduce the horizontal shedding of *M. synoviae* field isolates type C in outbreak areas as the results of immunization with live *M. gallisepticum* vaccines^35,37,38^.

Moreover, the PCR-RFLP assay was developed for detecting *M. synoviae* MS-H vaccine strain and differentiating it from *M. synoviae* non-vaccine strains circulating in Thailand, including *M. synoviae* WVU 1853 strain, Thai *M. synoviae* types C, E, and L. Based on the restriction enzyme map analysis tool on the Genescript webpage, the full-length amplicons and the two digested fragments of partial *vlhA* gene would be 350–400 bp and 100–250 bp, respectively. Besides, *M. synoviae* MS-H vaccine strain, whether from vaccine product, a few passages, or vaccinated chickens, was expectedly presented the specific two digested fragments size 100 and 134 bp, while other *M. synoviae* non-vaccine isolates; *M. synoviae* WVU 1853 strain, Thai *M. synoviae* field strains identified as types C, E, and L; were probably presented the specific two digested fragments size 130 and 160–210 bp. Consequently, the present study provided the first validated information of using the PCR-RFLP assay for detecting *M. synoviae* MS-H vaccine strain and differentiating it from non-vaccine strains circulating in Thailand; *M. synoviae* WVU 1853 strain, Thai *M. synoviae* field isolate types E and L. Thai *M. synoviae* isolate AHRU2020CU1323 (Fig. 1d, Lane 21) showing the specific two digested fragments size 100 and 134 bp of *M. synoviae* MS-H strain (Fig. 1d, Lane 20) might be spread horizontally from other vaccinated chicken in the same areas of high commercial farm density and heavy poultry traffic^39,40^. Therefore, the PCR-RFLP assay could be a convenient assay for evaluation of quality or uniformity of vaccination with live MS-H vaccine and could be further verified with other *M. synoviae* field strains recently circulating in other countries to affirm the differentiating efficacy and to fulfill the information of using vaccination to control *M. synoviae* disease^2–8,10,35,36,41^.

Furthermore, medication which is primarily performed with mycoplasma-susceptible antimicrobials inhibiting protein synthesis of mycoplasma organism^5,8,11^ is the common practice in the poultry industry due to an obvious efficacy in improving production performance of *M. synoviae* infected flocks by reducing clinical signs or economic losses^3–8,10,42^. Although the susceptible antimicrobials could reduce the populations of *M. synoviae* in respiratory tract like *M. gallisepticum*, could decrease the risks of horizontal transmission^43^, and could reduce the vertical transmission via eggs^42^; however, the long-term intensive medication, predisposing the antimicrobial resistance problems, is not economically acceptable in the infected poultry flocks^6,7,35^. The current antimicrobial resistance issues has influenced the practical medication in poultry industry, including the requirement of prescription from poultry veterinarian and the reliable susceptible profiles of *M. synoviae* field isolates^12,13^.

In this study, mycoplasma-susceptible antimicrobials commonly used in poultry industry^5,7,8,11^ consisting of tetracyclines (oxytetracycline and doxycycline), macrolides (tylosin, tilmicosin, tylvalosin), lincosamides (lincomycin), quinolones (enrofloxacin) and pleuromutilins (tiamulin); were determined MIC values by using the liquid method because of its simplicity and convenience compared with the agar or solid method^29^. This study provided the in vitro antimicrobial sensitivity information of each type of Thai *M. synoviae* isolates. Although antibiotic resistance levels are difficult to compare with the past antibiotic resistance levels, the present antimicrobial susceptibility profiles of *M. synoviae* field isolates could be reliable evidence for poultry veterinarian to make the prescription and design a suitable medication strategy for controlling *M. synoviae* problem in each farms^12,13^.

Although an susceptibility to tylosin, tilmicosin, tylvalosin, and tiamulin represented the effective antimicrobials and the attractive drug of choice in poultry farms, however, tetracyclines; doxycycline and oxytetracycline; could also perform a good activity against Thai *M. synoviae* isolates like a previous study in *M. gallisepticum* isolates showing good susceptibility to oxytetracycline^44^ and doxycycline^45^. In addition, the resistance to enrofloxacin in this study could notify that Thai *M. synoviae* isolates might be able to develop antimicrobial resistance against quinolones similar to the previous study in *M. gallisepticum*^44,45^.

In this study, the tested *M. synoviae* isolates were only second or third passage, so the number of microbial passages did not affect the resistant test. It is unlikely that the resistant test was induced during the microbial passages, which could lead to the selection of resistant mutants^46^. Therefore, antibiotic resistance development needs further studies about the history of antibiotics usage in poultry farms, including the effect of frequent or long-period usage.

In this study, some Thai *M. synoviae* isolates; AHRU2020CK0301, AHRU2020CK0305, and AHRU2020CK0404; were presented the final MIC value (at 0.625 μg/mL) of tilmicosin higher than other isolates; and some field isolates; AHRU2020CU1323 and AHRU2020CU1409; could perform the lower final MIC value than other isolates. In addition, the emergence of few isolates with high MIC values could have an asymmetrically strong influence on the MIC_50_ and MIC_90_ values^47^ as the difference between MIC_50_ and MIC_90_ values in doxycycline, oxytetracycline, tylosin, tilmicosin, tylvalosin, and tiamulin. Besides, the presence of a mixed population with different MIC levels in the field may contribute to resisting antimicrobials when a minor component with a high MIC level is growing with longer time of incubation as evident in fluoroquinolones^48^. However, in vitro resistance might also be the result of multi-step mutation affecting the permeability of cells, the uptake of the drug, and the binding to the ribosomes. Therefore, the different MIC levels among respiratory tract origin isolates and articular joint origin isolates in this study may be predisposed by the competence of tissues origin isolates, including ability to survive in the environment, ability to subsequent re-infection of chickens, ability to live in the respiratory organ or synovial tissue and ability to invade host cells for long periods or to reach subcellular fractions which antimicrobials would not be active^49^.

Due to the strong resistance to enrofloxacin of Thai *M. synoviae* field isolates in this study, in addition to studying the effect of many microbial passages, enrofloxacin-resistance mutants associated with alteration of the genes coding for DNA gyrase and topoisomerase IV should be further studied to determine the amino acid substitution^49^. Besides, the quinolone resistance-determining regions (QRDR) of DNA gyrase gene could be presented in the *gyrA* and *gyrB* genes, while QRDR of topoisomerase IV could show in *parC* and *parE* genes^49^.

In conclusion, in addition to recently defining the genetic characterization of Thai *M. synoviae* isolates types E and L by sequence analysis of partial *vlhA* gene, this study presented the validation of using the PCR-RFLP assay as the convenient diagnostic tool for strain differentiation, and provided the antimicrobial susceptibility profiles of current Thai *M. synoviae,* which are necessary to improve *M. synoviae* control strategies for poultry flocks in Thailand.

## Supplementary Information

Supplementary Information.

## Data Availability

The datasets, including sequence data generated and analyzed during this study, are available at the NCBI Nucleotide (https://www.ncbi.nlm.nih.gov/nuccore); see Table 2 for sample accession numbers.
