# Supplementary material for: Molecular characterization and antimicrobial susceptibility profiles of Thai Mycoplasma synoviae isolates
Source: Sci Rep. 2023 Feb 3;13:2002. doi: 10.1038/s41598-023-29266-9 (PMC9898534; doi:10.1038/s41598-023-29266-9)
Supplement: Supplementary file 1 — Supplementary Information. [file 41598_2023_29266_MOESM1_ESM.docx]

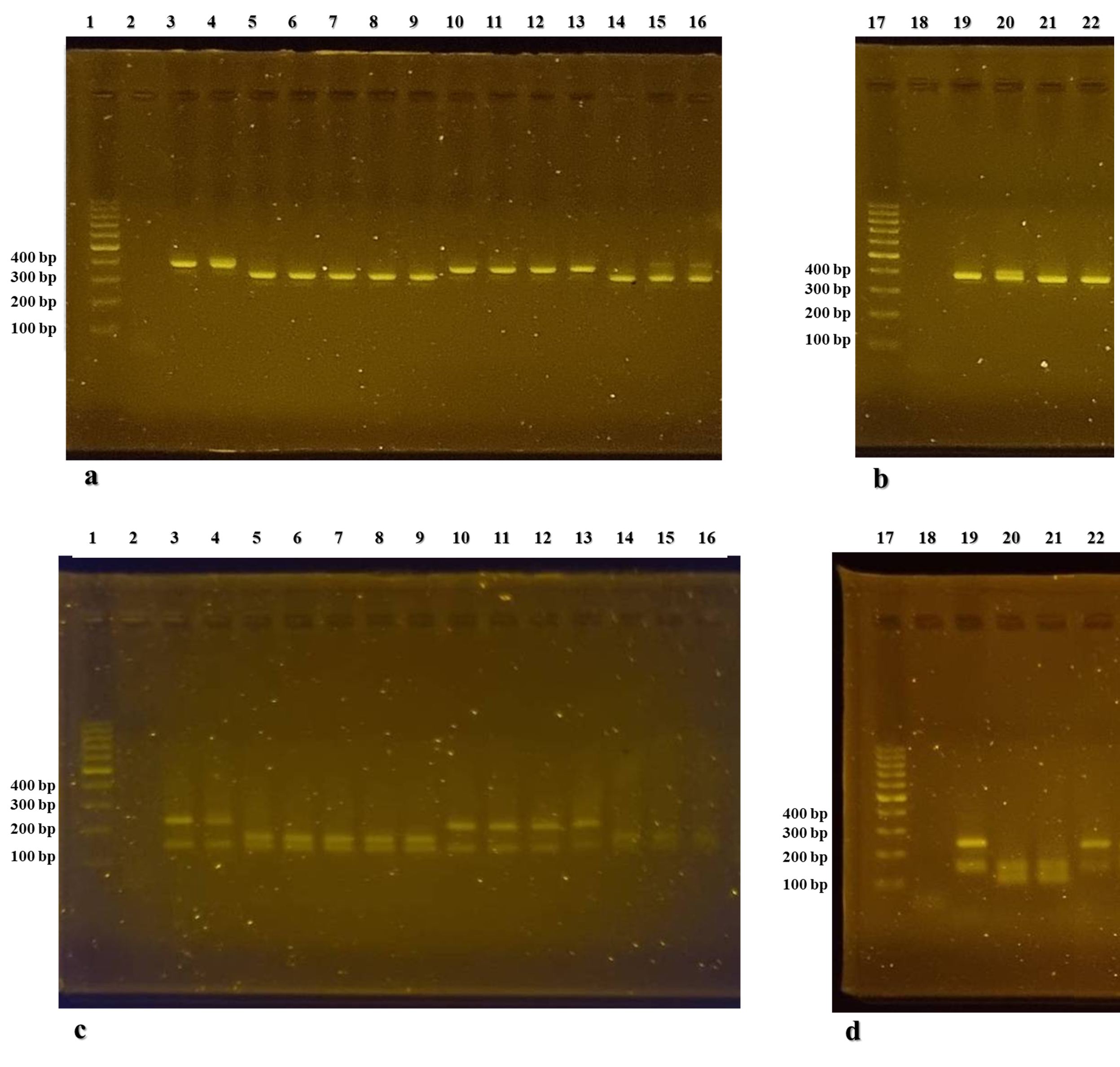


**Supplementary Figure 1**: The original, unprocessed version of electrophoresis gel with membrane edges visible demonstrating PCR products from *M. synoviae* isolates consisting of the full length of partial *vlhA* gene amplicons size of 350-400 bp (a, b) and the two digested fragments of partial *vlhA* gene amplicons size of 100-200 bp (c, d).

Lane 1 and 17, 1000 bp DNA ladder;

Lane 2 and 18, *M. gallisepticum* S6 strain as negative control;

Lane 3 and 19, *M. synoviae* WVU 1853 strain as positive control;

Lane 4, *M. synoviae* MS-1 strain;

Lane 5, AHRU2020CK0615; Lane 6, AHRU2020CK0301; Lane 7, AHRU2020CK0305;

Lane 8, AHRU2020CK0404; Lane 9, AHRU2020CK0709;

Lane 10, AHRU2020CU1401; Lane 11, AHRU2020CU1409; Lane 12, AHRU2020CU1505;

Lane 13, AHRU2015CU2802;

Lane 14, AHRU2018CK0301; Lane 15, AHRU2020CU1104; Lane 16, AHRU2020CU1101;

Lane 20, *M. synoviae* MS-H vaccine strain

Lane 21, AHRU2020CU1323;

Lane 22, AHRU2020CK1206;
